# *SCARB2* variants and glucocerebrosidase activity in Parkinson’s disease

**DOI:** 10.1038/npjparkd.2016.4

**Published:** 2016-03-10

**Authors:** Roy N Alcalay, Oren A Levy, Pavlina Wolf, Petra Oliva, Xiaokui Kate Zhang, Cheryl H Waters, Stanley Fahn, Un Jung Kang, Christopher Liong, Blair Ford, Pietro Mazzoni, Sheng Kuo, Amelie Johnson, Lan Xiong, Guy A Rouleau, Wendy K Chung, Karen S Marder, Ziv Gan-Or

**Affiliations:** 1 Department of Neurology, College of Physicians and Surgeons, Columbia University Medical Center, New York, NY, USA; 2 Taub Institute for Research on Alzheimer's Disease and the Aging Brain, College of Physicians and Surgeons, Columbia University Medical Center, New York, NY, USA; 3 Biologics Structural and Functional Research, Biopharmaceutics Development, Genzyme, a Sanofi company, Framingham, MA, USA; 4 Département de médicine, Université de Montréal, Montréal, QC, Canada; 5 Laboratoire de neurogénétique, Centre de recherche, Institut universitaire en santé mentale de Montréal, Montréal, QC, Canada; 6 Département de Psychiatrie, Université de Montréal, Montréal, QC, Canada; 7 Department of Neurology and Neurosurgery, McGill University, Montréal, QC, Canada; 8 Montréal Neurological Institute & Hospital, McGill University, Montreal, QC, Canada; 9 Department of Human Genetics, McGill University, Montreal, QC, Canada; 10 Department of Pediatrics and Medicine, College of Physicians and Surgeons, Columbia University Medical Center, New York, NY, USA; 11 Gertrude H. Sergievsky Center, College of Physicians and Surgeons, Columbia University, New York, NY, USA

## Abstract

Mutations in glucocerebrosidase (*GBA*) are a common risk factor for Parkinson's disease (PD). The scavenger receptor class B member 2 (*SCARB2*) gene encodes a receptor responsible for the transport of glucocerebrosidase (GCase) to the lysosome. Two common SNPs in linkage disequilibrium with *SCARB2*, rs6812193 and rs6825004, have been associated with PD and Lewy Body Disease in genome-wide association studies. Whether these SNPs are associated with altered glucocerebrosidase enzymatic activity is unknown. Our objective was to determine whether *SCARB2* SNPs are associated with PD and with reduced GCase activity. The *GBA* gene was fully sequenced, and the *LRRK2* G2019S and *SCARB2* rs6812193 and rs6825004 SNPs were genotyped in 548 PD patients and 272 controls. GCase activity in dried blood spots was measured by tandem mass spectrometry. We tested the association between *SCARB2* genotypes and PD risk in regression models adjusted for gender, age, and *LRRK2* G2019S and *GBA* mutation status. We compared GCase activity between participants with different genotypes at rs6812193 and rs6825004. Genotype at rs6812193 was associated with PD status. PD cases were less likely to carry the T allele than the C allele (OR=0.71; *P*=0.004), but GCase enzymatic activity was similar across rs6812193 genotypes (C/C: 11.88 μmol/l/h; C/T: 11.80 μmol/l/h; T/T: 12.02 μmol/l/h; *P*=0.867). Genotype at rs6825004 was not associated with either PD status or GCase activity. In conclusion, our results support an association between *SCARB2* genotype at rs6812193 and PD, but suggest that the increased risk is not mediated by GCase activity.

## Introduction

Heterozygous mutations and variants in glucocerebrosidase (*GBA*) are present in 3–5% of individuals with Parkinson’s disease (PD).^1,2^ The scavenger receptor class B member 2 (*SCARB2*) gene encodes a protein, lysosome membrane protein 2 (LIMP-2), that transports β-glucocerebrosidase (GCase) from the endoplasmic reticulum through the Golgi apparatus and endosomes to the lysosome.^3^ Homozygous mutations in *SCARB2* cause a rare form of progressive myoclonic epilepsy, action myoclonus-renal failure.^3^ Affected patients have significantly reduced GCase activity (7 nmol/mg protein per h compared with 15 nmol/mg protein per h in controls),^3,4^ that is in the range observed for carriers of Gaucher disease (*GBA* heterozygotes). Two common single-nucleotide polymorphisms (SNPs), rs6812193 (refs 5,6; 5′ of *SCARB2*) and rs6825004^7,8^ (intron in *SCARB2*), have been shown to be associated with PD^5–7,9^ and Dementia with Lewy Bodies^8^ in several genetic studies, including large genome-wide association studies. Furthermore, given that homozygous mutations in *SCARB2* are associated with reduced GCase activity,^3^ it is possible that these SNPs modify the risk for PD via modulation of GCase activity. In this study, we tested the hypotheses that (1) these two SNPs would be associated with PD in a New York PD cohort; and (2) that protective variants are associated with higher GCase enzymatic activity as measured in dried blood spots when compared with carriers of the non-protective variant (e.g., if indeed rs6812193 is associated with lower PD risk via *SCARB2* then carriers of the protective nucleotide, T, will have higher GCase activity than carriers of the C allele).

## Results

Eight hundred and twenty participants were genotyped for the *SCARB2* SNPs, including 548 PD cases and 272 controls. Demographics and *GBA* and *LRRK2* mutation status are presented in Table 1. The genotype and allele frequencies of rs6812193 and rs6825004 are presented in Table 2. Genotype at rs6825004 was not associated with PD status in our cohort, nor was it associated with GCase activity (C/C genotype 11.75 μmol/l/h; C/G genotype 11.94 μmol/l/h; G/G genotype: 11.99 μmol/l/h; *P*=0.703. This comparison included PD cases and controls and excluded *GBA* and *LRRK2* p.G2019S mutation carriers). Genotype at rs6812193 was associated with PD status. The C allele was associated with a higher risk of PD (Table 2; *P*=0.004) consistent with previous reports.^5,6^ However, rs6812193 genotype was not associated with differential GCase activity (C/C: 11.88 μmol/l/h; C/T: 11.80 μmol/l/h; T/T: 12.02 μmol/l/h; *P*=0.867. This comparison included PD cases and controls and excluded *GBA* and *LRRK2* p.G2019S mutation carriers).

## Discussion

Our findings support previous reports that the genotype at rs6812193 SNP is associated with PD. This SNP, which is in linkage disequilibrium with the *SCARB2* gene, was found in association with PD in two large multi-ethnic genome-wide association studies (OR=0.84; *P*=7.6×10^−10^ and OR=0.907; *P*=2.95×10^−11^)^5,9^ and in a German association study (OR=0.86; *P*=0.02).^6^ However, the association was not replicated in a Chinese^10^ or a Greek cohort,^11^ likely due to either population differences or insufficient power. Here, we confirm the association in a clinic based New York cohort, which consists of 40% AJ PD patients. An association between the *SCARB2* gene and PD was hypothesized to be due to differential trafficking of GCase to the lysosome.^4^ In the current study we hypothesized that if indeed rs6812193 is associated with PD risk via *SCARB2,* then carriers of the protective nucleotide (T) should have higher GCase activity than carriers of the PD susceptibility allele (C). We have previously shown in dried blood spots that heterozygous *GBA* carriers have lower GCase activity than non-carriers (by ~33%), and that idiopathic PD cases have slightly lower GCase activity (by ~5%) than controls.^12^ Our current findings do not support the link between rs6812193 and PD through a mechanism of reduced GCase activity (at least not as measured in dried blood spots). There are several possible explanations for our findings. First, rs6812193 is associated with PD via a different gene/mechanism not linked to *SCARB2* or GCase. This explanation is supported by a prior study showing that variants in this SNP do not correlate with LIMP-2 messenger RNA or protein levels in leukocytes from a small sample of controls.^13^ Second, rs6812193 is associated with PD through *SCARB2,* but its biological effect is not mediated by GCase activity. Finally, rs6812193 is associated with PD through *SCARB2* and GCase pathways but this effect is either present in selected tissues (e.g., brain) and not leukocytes, or it involves altered intracellular routing and localization of GCase that is not apparent in the DBS assay.

In addition, we were not able to replicate the association between rs6825004 and PD. This association was reported in a Greek cohort^7^ and was also reported in a Dementia with Lewy Body association study, but not in other PD genome-wide studies.^8^ It is possible that we did not observe the association because our cohort did not include Dementia with Lewy Body cases.

In summary, we provide evidence that *SCARB2* is associated with PD although the mechanism remains unknown. Full sequencing of *SCARB2* in PD cohorts and correlation of PD risk with GCase activity may help to clarify these associations further.

## Materials and methods

### Participants and clinical evaluation

Participants in the ‘SPOT’ study included PD patients and genetically unrelated controls (mostly spouses) from the Center for Parkinson’s Disease at Columbia University Medical Center in New York, NY, recruited between 2010 and 2014.^14^ The cohort has been previously described.^12^ In brief, a blood sample and the data on demographics, medical history, medication, PD family history,^15^ the Unified Parkinson’s Disease Rating Scale (UPDRS) in the ‘on’ state and the Montreal Cognitive Assessment (MoCA)^16^ were collected from consecutive PD cases, as defined by the United Kingdom PD brain bank criteria (except that we did not exclude cases with a family history of PD).^17^ A blood sample was collected from genetically unrelated control individuals, mostly spouses. All study procedures were approved by the Columbia University IRB, and all participants signed informed consent.

### Genotyping of *GBA* and *SCARB2*

All study participants were fully sequenced for *GBA* mutations and genotyped for the *LRRK2* p.G2019S mutation as previously described.^12^ Two *SCARB2* SNPs were genotyped by TaqMan SNP genotyping assays, rs6812193 and rs6825004 (assay IDs: C__31139749_10 and C___1129894_20, respectively) following the manufacturer’s instructions, and the genotypes were called using QuantStudio™ 7 Flex Real-Time PCR System and Software (Applied Biosystems, Foster City, CA, USA).

### GCase activity assay

Dried blood spots were obtained as previously described.^12,18,19^ In brief, 75 μl of blood was ‘spotted’ on a filter paper and dried at room temperature for at least 4 h. GCase activity was measured at Sanofi Genzyme laboratories using a previously published protocol as part of a multiplex assay together with four additional lysosomal enzymes.^20^ Activity was expressed as micromoles of product per liter of whole blood per hour (μmol/l/h). All Sanofi Genzyme scientists were blinded to PD and genetic status.

### Statistical analysis

Demographics, frequency of *GBA* variants, *LRRK2* p.G2019S mutations, rs6812193 and rs6825004 genotypes were compared between PD cases and controls using the Student *t-*test for continuous variables, and the chi-square and Fisher’s exact tests for categorical variables. We used logistic regression analyses to test whether the SNPs (predictors) are associated with PD status (outcome) in models adjusted for age, gender, and *GBA* and *LRRK2* mutation status.

To test the association between GCase activity and the rs6812193 and rs6825004 genotypes, we first compared GCase enzymatic activity by genotypes in the entire sample (including PD cases and controls), and then in PD cases and controls separately. Analyses of GCase activity were repeated excluding all carriers of *GBA* variants previously associated with reduced GCase activity and the *LRRK2* p.G2019S mutation which was previously shown to be associated with increased GCase activity.^12^

Analyses were performed using SPSS Statistics version 21.0 software (Chicago, IL, USA).

## Figures and Tables

**Table 1:** Demographics, *GBA* and *LRRK2* mutation status in PD cases and controls

	*PD cases (*n*=548)*	*Controls (*n*=272)*	P*-value*
Mean age in years, (s.d.)	66.0 (10.5)	65.3 (9.5)	0.339
Percent male, (*n*)	64.4% (353)	34.9% (95)	<0.001
Percent with at least one Ashkenazi Jewish grandparent, (*n*)	43.8% (240)	39.9% (107)	0.521
Percent with family history of PD in first-degree relative, (*n*)	17.5% (96)	4.8% (13)	<0.001
Carriers of *LRRK2* p.G2019S	7.3% (40)	0.7% (2)	<0.001
Carriers of any *GBA* variants^a^	16.6% (91)	6.6% (18)	<0.001
Education in years, (s.d.)	16.6 (2.9)	16.7 (2.7)	0.691
UPDRS- part III, (s.d.)	17.9 (10.6)	1.0 (1.8)	<0.001
MoCA, (s.d.)	25.3 (3.7)	27.0 (2.2)	<0.001
Mean PD age-at-onset, (s.d.)	59.2 (11.6)		
Levodopa equivalent daily dose in mg, (s.d.)	535 (461)		

Abbreviations: MoCA, Montreal Cognitive Assessment; PD, Parkinson’s disease; UPDRS, Unified Parkinson’s Disease Rating Scale.

a Including heterozygotes, homozygotes and compound heterozygotes of GBA mutations and variants.^11^

**Table 2:** Association between rs6812193 and rs6825004 genotypes and Parkinson’s disease

*Status*	*Controls (*n*=272)*		*PD Cases (*n*=548)*		P*-value for PD risk*	*OR for PD (95% CI)* a
	N *(%)*	*GCase enzymatic activity* b *μmol/l/h (s.d.)*	N *(%)*	*GCase enzymatic activity* b *μmol/l/h (s.d.)*		
*rs6812193*						
C/C	115 (42.3%)	11.97 (3.31)	273 (49.8%)	11.82 (3.16)	0.057	Reference
						
C/T	123 (45.2%)	11.96 (2.90)	229 (41.8%)	11.70 (3.09)		0.52 (0.30–0.89); *P*=0.018
T/T	34 (12.5%)	12.07 (4.43)	46 (8.4%)	11.98 (3.10)		0.62 (0.36–1.07); *P*=0.086
C	353 (64.8%)		775 (70.7%)		0.0175	0.71 (0.56–0.90); *P*=0.004^c^
T	191 (35.2%)		321 (29.3%)			
						
*rs6825004* d						
C/C	125 (46.1%)	11.94 (3.35)	243 (44.4%)	11.63 (2.88)	0.562	Reference
						
C/G	115 (42.4%)	11.90 (3.22)	229 (41.9%)	11.97 (3.42)		1.03 (0.74–1.45); *P*=0.833
G/G	31 (11.4%)	12.42 (3.33)	75 (13.7%)	11.76 (3.04)		0.79 (0.48–1.30); *P*=0.788
C	365 (67.3%)		715 (65.3%)		0.438	1.17 (0.93–1.47); *P*=0.182^c^
G	177 (32.7%)		379 (34.7%)			

Abbreviations: CI, confidence interval; OR, odds ratio; PD, Parkinson's disease.

a OR calculated in models adjusted for age, gender, *LRRK2* and *GBA* mutation status.

b There was no difference in GCase activity among the genotypes, either in controls or in PD cases. Values presented here after excluding *GBA* and *LRRK2* p.G2019S carriers.

c Model included sex, age, rs6812193 genotype, and rs6825004 genotype.

d rs6825004 was missing on one PD and one control participant.
